# Research Progress on the Application of Radiomics and Deep Learning in Liver Fibrosis

**DOI:** 10.3390/jimaging12020082

**Published:** 2026-02-15

**Authors:** Yi Dang, Wenjing Li, Zhao Liu, Junqiang Lei

**Affiliations:** 1 Department of Radiology, The First Hospital of Lanzhou University, Lanzhou 730000, China; duaiad139@163.com (Y.D.); l15236636689@163.com (W.L.); 2 Intelligent Imaging Medical Engineering Research Center of Gansu Province, Lanzhou 730000, China

**Keywords:** radiomics, deep learning, multimodal fusion, liver fibrosis

## Abstract

Liver fibrosis (LF) represents a crucial intermediate stage in the pathological progression from chronic liver disease to cirrhosis and hepatocellular carcinoma. Early and accurate diagnosis is of vital importance for the intervention treatment of diseases and the improvement of prognosis. Traditional liver biopsy, long regarded as the diagnostic gold standard, remains associated with several notable limitations such as invasiveness, sampling errors and inter-observer variability. Lately, as artificial intelligence (AI) technology progresses swiftly, radiomics and deep learning (DL) have risen to prominence as non-invasive diagnostic instruments, showing significant potential in the LF diagnostic evaluation. This review summarizes the latest advancements in radiomics and DL for LF diagnosis, staging, prognosis prediction and etiological differentiation. It also analyzes the application value of multimodal imaging modalities, including magnetic resonance imaging (MRI), computed tomography (CT) and ultrasound in this field. Despite ongoing challenges in model generalization and standardization, improved model interpretability, technological integration and multimodal fusion, the continuous advancement of radiomics and DL technologies holds promise for AI-driven imaging analysis strategies. These approaches aim to integrate multiple clinical monitoring methods, overcome obstacles in the early LF diagnosis and treatment and provide new perspectives for precision medicine of this disease.

## 1. Introduction

Liver fibrosis (LF) represents an abnormal reparative response of the liver to various chronic insults, including viral, toxic, metabolic and autoimmune diseases. It is primarily characterized by excessive deposition of extracellular matrix (ECM) and the formation of scar tissues [1]. This can disrupt liver structure and impair organ function, altering blood flow within the liver, and may ultimately result in cirrhosis or liver cancer [2]. It also poses a significant public health challenge, imposing a heavy economic burden and mortality risk worldwide [3]. Current epidemiological data suggest that the cumulative number of cirrhosis patients in the United States alone may reach approximately 2.2 million. Between 2010 and 2021, the annual mortality rate from cirrhosis rose from 14.9 per 100,000 people to 21.9 per 100,000 people [4]. Liver transplantation offers a life-saving treatment for cirrhosis and hepatocellular carcinoma patients, but organ demand far exceeds supply [5]. Moreover, early-stage LF can regress or even be reversed with timely and effective intervention. Therefore, early detection and accurate assessment of LF status hold immense clinical significance.

At present, liver biopsy remains widely regarded as the gold standard for diagnosing and assessing the degree of LF. Nevertheless, as an invasive procedure, it presents numerous limitations, including its invasive property, variability in sample collection and unsuitability for large-scale screening [6]. These factors have prompted clinicians to urgently seek non-invasive approaches for early diagnosis and staging of LF. Non-invasive modalities, exemplified by conventional imaging studies (magnetic resonance imaging (MRI), computed tomography (CT) and ultrasound), can diagnose advanced LF and cirrhosis based on hepatic morphological changes. However, in the early stages of LF, these imaging features are often subtle, making it difficult to accurately assess the extent of disease progression [7]. Consequently, developing accurate non-invasive methods for the early assessment of LF is of great significance for the accurate diagnosis, continuous monitoring and individualized therapy of liver diseases.

**Figure 1:**
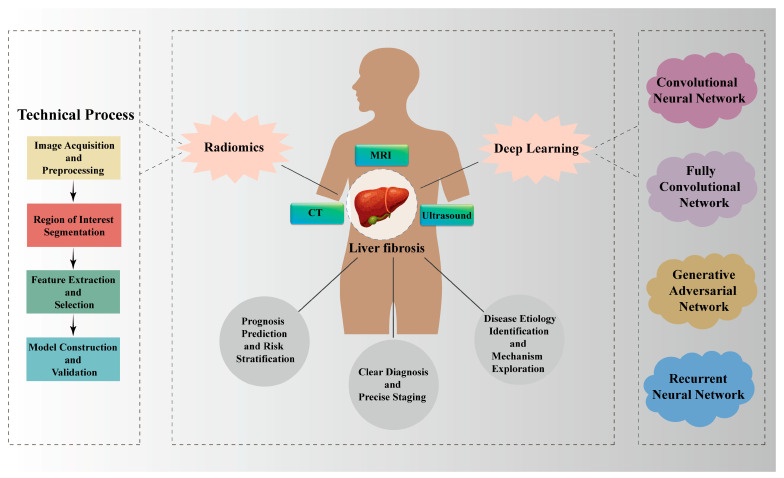
**Applications of Radiomics and Deep Learning in Liver Fibrosis.** Radiomics and DL technologies play a role in the precise diagnosis and staging of LF, prognosis prediction and risk stratification, as well as etiological differentiation and mechanism exploration through multiple imaging modalities (MRI, CT and ultrasound).

The emergence of radiomics offers a new approach to solving this challenge [8]. By extracting abundant quantitative features from medical imaging data, radiomics enables comprehensive data mining and in-depth analytical processes. By capturing microscopic textures and functional information invisible to the human eye, it supplements the assessment of clinical indicators for various diseases [9]. These high-throughput imaging features can reflect pathophysiological changes within the liver, such as inflammation and fibrosis [10]. With advances in medical imaging technology and the development of artificial intelligence (AI) algorithms, data model analyses represented by radiomics and deep learning (DL) techniques have been increasingly adopted in the medical domain [11], which is profoundly transforming traditional imaging monitoring methods, offering an innovative tool for the non-invasive and accurate evaluation of LF (Figure 1). To facilitate a comprehensive understanding of this rapidly evolving field, this review is structured as follows: Section 2 delineates the technical workflows and distinct characteristics of radiomics and DL, providing a comparative analysis of their strengths and limitations in LF assessment. Section 3 systematically examines specific applications across different imaging modalities (MRI, CT and ultrasound), highlighting how each technique contributes unique dimensional information to the evaluation. Section 4 extends beyond diagnosis to explore advanced clinical scenarios, specifically focusing on prognosis prediction, risk stratification and the exploration of etiological mechanisms. Finally, Section 5 critically discusses current challenges regarding standardization and interpretability, and offers a prospective outlook on future developments such as multimodal fusion and explainable AI, thus providing strategies to accelerate the clinical translation of these innovative technologies in LF.

## 2. Radiomics and Deep Learning in Liver Fibrosis Assessment

### 2.1. Technical Workflow of Radiomics

Radiomics represents an emerging methodological framework that systematically extracts a large set of quantitative radiological features from medical images to support data analysis and predictive modeling [12]. Its technical workflow encompasses multiple stages, forming a comprehensive analytical chain that provides objective support for clinical diagnosis and treatment. First of all, image acquisition serves as the foundation, where medical images with quantitative data are obtained through various imaging modalities (MRI, CT and ultrasound) for subsequent feature extraction [13]. However, differences in imaging equipment, scanning parameters and protocols used across medical institutions may lead to variations in omics features [14]. Therefore, image standardization and preprocessing are necessary to enhance image quality. Nevertheless, the application of standardization protocols in radiomics research still requires improvement [15]. Next, it is necessary to delineate the region of interest (ROI) on the image, which serves as a prerequisite for feature extraction [9]. Traditionally, two-dimensional ROIs have been delineated using manual or semi-automated methods: manual segmentation is characterized by substantial time consumption and susceptibility to inter-observer bias [16], while semi-automated segmentation based on various algorithms requires experienced radiologists to assist with corrections [17]. Currently, the whole-volume ROI method is primarily used to segment the entire liver [18,19], thereby providing additional dimensions for feature extraction in the images and better reflecting the full picture of liver lesions.

Feature extraction and selection form the core of radiomics. Numerous quantitative features could be extracted from processed images, including first-order statistical features (e.g., energy, entropy, uniformity, etc.), texture features (such as gray-level co-occurrence matrix features), morphological features and higher-order wavelet features [20]. However, only some of the extracted features are relevant to the prediction model, whereas others might be inconsequential or result in overfitting [21]. As a consequence, feature selection including filtering, wrapping, embedding and combination is necessary, followed by uniformization processing [20]. Model building and validation constitute the final stage, where predictive models are constructed using diverse machine learning algorithms (support vector machines, random forests, logistic regression, etc.), and model performance is evaluated through methods like cross-validation and external validation [9]. The training and testing datasets used in the study were configured with internal and external validation sets based on the optimization requirements of algorithm parameters and hyperparameters for specific tasks. Decisions on the best dimensionality reduction and regularization choices were based on the most effective outcomes noted in these sets [12].

Currently, radiomics has demonstrated significant advantages in the assessment of LF. Wang et al. crafted a radiomics model to predict fibrosis using liver-enhanced CT images. They found that the R-LF and R-score, derived from the training cohort data, attained an area under the curve (AUC) of 0.84–0.90, with a predictive accuracy of 82–86% for LF staging in the validation cohort. This confirms that radiomics analysis of enhanced CT imaging features enables more accurate staging of LF [22]. In a recent study targeting patients with fibrosis caused by chronic hepatitis B virus, the research team developed a predictive model using multi-sequence liver MRI technology in conjunction with eight machine learning algorithms. From 1050 quantitative imaging features, they identified 58 with the highest predictive value. The resulting support vector machine model achieved AUC values of 0.94 (training set) and 0.93 (testing set), which were significantly outperforming traditional clinical indicators [23]. This showcases the effective creation of a radiomics framework based on multi-sequence liver MRI, potentially improving risk assessment and treatment for LF patients. In summary, these studies confirm that radiomics can capture the spatial heterogeneity and staging of LF, providing diagnostic information that surpasses traditional imaging assessments.

### 2.2. Technical Characteristics of Deep Learning

As an essential component of machine learning, DL simulates the hierarchical information processing mechanisms of the human brain by constructing multi-layer neural networks for handling extensive data sets [24]. Unlike traditional radiomics approaches that rely on manual design and evaluation, DL possesses end-to-end learning capabilities, which empower it to automatically extract discriminative features directly from raw image data to achieve high classification performance [25]. This characteristic makes DL particularly suitable for the complex disease progression, such as LF classification and evaluation in medical imaging [26].

In liver disease assessment, commonly used DL architectures include convolutional neural network (CNN), fully convolutional network (FCN), recurrent neural network (RNN) and generative adversarial network (GAN), which have gained prominence in recent years [27]. These models extract image features step by step through multiple layers of nonlinear transformations: shallow networks capture local details (like texture or edges), while deep networks identify more complex pathological patterns (such as jagged boundaries of cancer or nodular changes) [28]. Researchers have developed a liver magnetic resonance elastography (MRE) analysis method based on automated CNN. They found that the AUC range for CNN-based analysis was between 0.89 and 0.93, while the AUC for manual ROI-based analysis was between 0.87 and 0.93 (*p* = 0.23–0.75). Moreover, the CNN-based method demonstrated excellent differentiation performance in external testing for fibrosis stages determined by histology [29]. This result validates the practicality of CNN-based analysis, potentially diminishing the need for expert image analysts. Another study demonstrated that a DL model based on high-frequency ultrasound images exhibited superior diagnostic performance across all LF stages in chronic hepatitis B patients, particularly in distinguishing advanced fibrosis with an accuracy of 0.93 (*p* < 0.01) [30]. This model outperformed other commonly used non-invasive assessment methods, suggesting significant potential clinical value.

Another core strength of DL lies in its multimodal data fusion capabilities [31]. Assessing LF often requires integrating imaging, clinical parameters and pathology information, while DL models effectively fuse multi-source data (like various CT images, etiology and laboratory test results), by comparing features extracted through CNN, utilizing shared databases and employing standardized reporting. This significantly enhances the model’s capacity to generalize and improves DL outcomes [26]. For example, the combined model (AutoFibroNet) constructed by integrating DL features, clinical indicators and manual features from conventional image processing methods demonstrated strong discriminatory capability on the training set: for fibrosis stages F0 to F4, its Area Under the Receiver Operating Characteristic Curve (AUROC) values were 1.00, 0.99, 0.98 and 0.98, respectively. Across the two validation cohorts, AutoFibroNet achieved AUROC values for F0–F4 fibrosis stages of 0.99, 0.83, 0.80, 0.90, 0.88 (Cohort 1) and 1.00, 0.83, 0.80, 0.94, 0.91 (Cohort 2), respectively, which indicates that it has good discrimination ability across different cohorts [32]. However, achieving an AUROC of 1.00 in medical imaging research is extremely rare. Further studies involving larger and more diverse populations are still required to validate the robustness and generalizability of this model. Moreover, DL demonstrates high diagnostic efficacy and stability in LF applications. Research has shown that the transfer learning radiomics model combining grayscale modality and elastography ultrasonographic images could accurately achieve LF grading (with AUC values of 0.950 for S4, 0.932 for ≥S3 and 0.930 for ≥S2, *p* < 0.05) [33]. This study maintains high accuracy while enabling non-invasive detection. The stability of DL is partly due to its anti-interference capabilities. For instance, through data augmentation techniques (like image rotation and flipping) and regularization methods, the model could overcome issues like uneven image quality in medical imaging caused by texture and non-stationary noise [34]. This provides a more reliable data foundation for extracting robust radiomics features. Further studies are needed to explore its stability in LF applications.

In recent years, self-supervised DL has also made progress in the study of hepatic focal lesions [35]. It extracts valuable supervisory information from vast and unlabeled medical datasets through predefined tasks, thereby training neural networks to guide model training [31]. These methods reduce reliance on extensive quantities of labeled data, providing new tools for exploring the mechanisms of LF. Compared to machine learning, DL demonstrates superior overall performance in LF diagnosis. A meta-analysis encompassing 106 studies revealed that DL models achieved an overall AUC of 0.875 for diagnosing LF, outperforming machine learning (AUC = 0.826). Among these, CNN and ResNet50 models demonstrated superior effectiveness, with AUC values reaching 0.917 and 0.960 respectively [36]. The advantage of DL stems from its ability to process high-dimensional data. However, it typically requires large-scale labeled datasets and efficient computational resources, while exhibiting poor model interpretability. Therefore, further improvements are needed to enhance its interpretability.

In summary, DL demonstrates significant potential in non-invasive LF assessment through automated feature learning and multimodal integration. This not only improves diagnoses precision but also offers novel approaches for prognosis prediction and treatment monitoring. However, its clinical translation still requires addressing challenges such as data standardization, model generalization and result interpretation.

### 2.3. Comparison Between Radiomics and Deep Learning

As two mainstream technologies in medical image analysis, radiomics and DL each possess unique value and technical characteristics in LF assessment [37]. Specifically, first of all, there are differences in the technical principles and feature extraction methods: radiomics relies on manually designed feature extraction processes, and these features are modeled using conventional machine learning techniques like support vector machines and random forests. The process is highly dependent on feature extraction guided by domain knowledge [38]. In contrast, DL automatically extracts features from raw image data through multi-layer neural networks such as CNN, without requiring manual intervention [39]. This mechanism enables DL to uncover complex patterns that are difficult for the human eye to recognize, optimizing the non-invasive evaluation of LF on a large scale [26]. Secondly, there is a comparison between data requirements and model performance. DL models can automatically learn the features contained in the hidden layers of neural networks from image data, but their application requires a large amount of image data to support it [40]. For example, in a disease detection study based on radiomics and DL [41], researchers found that the radiomics model maintained an AUC value of 0.762 even under small sample conditions (24 samples) and exhibited a more stable learning curve. However, when the sample size reached 4000, the DL model yielded a high AUC of 0.996 and an accuracy of 0.960. Under small sample conditions, its performance exhibited significant variability (F1 score standard deviation ± 0.062). These results indicate that DL models demonstrate superior performance and enhanced scalability with the expansion of data accessibility, whereas radiomics models remain a viable option when data volumes are limited. The third point is the trade-off between interpretability and computational demands. The core of radiomics research lies in integrating imaging features with statistical models to achieve disease-related prediction and analysis through feature engineering and various machine learning algorithms. DL, on the other hand, leverages structures like CNNs to automatically extract image features, thereby achieving high classification performance. When combined with imaging techniques, both approaches demonstrate promising potential in identifying biological characteristics and predicting prognosis for liver diseases. However, the lack of model interpretability remains a key issue limiting their clinical application [24]. Therefore, further prospective, large-scale and multicenter studies are still needed to validate their reliability and advance clinical translation. In terms of computational resource requirements, radiomics models can run on conventional computing devices, making them easier to deploy in healthcare institutions with limited resources. However, as AI progresses swiftly and learning algorithms become more intricate, training DL models necessitates high-performance GPU clusters, demanding substantial computational power from hardware infrastructure [42]. Furthermore, the two technologies exhibit complementary strengths across different LF assessment scenarios: radiomics combined with traditional machine learning algorithms (logistic regression) may hold clinical value for early screening and preliminary evaluation of LF [43], while DL demonstrates greater advantages in precise staging and prognosis prediction for LF [44].

Traditional methods are unable to interpret the high-dimensional details brought about by advancements in imaging technology, but imaging genomics, under DL methods, can efficiently extract high-dimensional features from imaging data to assist in diagnosis [45]. Currently, the boundaries between radiomics and DL are becoming increasingly blurred, with a growing trend toward integration [26]. Combining radiomics with DL features enables the construction of more powerful predictive models. In summary, radiomics and DL are not mutually exclusive in LF assessment, rather, they can complement and integrate in practical applications. For instance, one could attempt to combine manually designed radiomic features with DL features to construct a fusion model. This hybrid approach retains the interpretability advantage of radiomics while leveraging the powerful feature learning capabilities of DL, jointly advancing the precision and clinical utility of non-invasive LF assessment. To further clarify the distinction between the methodologies discussed above, Table 1 provides a detailed comparison between the hand-crafted features used in radiomics and the learned features extracted by deep learning models.

## 3. Imaging Modalities and Data Characteristics

LF’s radiomics and DL analysis can be performed across multiple imaging modalities such as MRI, CT and ultrasound. Each imaging technique possesses distinct characteristics, providing complementary information dimensions for LF assessment [26].

### 3.1. Magnetic Resonance Imaging Sequences

MRI, with its multi-parameter and multi-sequence imaging capabilities, could reflect pathophysiological changes during LF from various perspectives and has emerged as a crucial instrument for the non-intrusive evaluation of this disease [46]. Typical conventional MRI sequences include T1-weighted imaging (T1WI), T2-weighted imaging (T2WI), in-phase/out-of-phase imaging, as well as diffusion weighted imaging (DWI) and contrast-enhanced scanning. These sequences reflect the pathological and physiological state of liver tissue from different perspectives [47]. Specifically, T1WI and T2WI form the foundation of liver MRI examinations. However, early fibrosis is confined to the periportal areas of the liver lobules, with minimal changes in tissue structure and stiffness. Consequently, traditional T1WI or T2WI face challenges in identifying these lesions [48]. As fibrosis progresses, morphological changes such as surface irregularities occur in the liver, and these alterations can be observed on both T1WI and T2WI [49]. Applications based on DL algorithms can automatically identify these subtle morphological changes, thereby improving the efficiency of early diagnosis [50]. Existing studies have shown that deep convolutional neural networks (DCNN)-based analysis of MRI sequences delivers AUC values of 0.85, 0.84 and 0.84 for stages F2, F3 and F4, respectively [51]. Additionally, DWI reflects changes in tissue microstructure by measuring the diffusion motion of water molecules [52]. Liver microcirculatory disorders may occur during the LF process, leading to edema within and around hepatocytes. As edema worsens and organelles are destroyed, hepatocyte necrosis may result. These pathological changes restrict the free diffusion of water molecules, causing a decrease in the apparent diffusion coefficient (ADC) value [53]. Zhu and his colleagues used a five-layer DCNN to analyze magnetic resonance ADC images. They found that the accuracy in staging chronic hepatitis B LF reached 88.13%, which was significantly higher than the traditional ADC value measurement (75.07%) [54]. However, the ADC value alone is not specific enough for assessing LF and still requires integration with other sequences for a comprehensive judgment.

MRE is currently the most accurate non-invasive imaging method for assessing LF, primarily evaluating fibrosis severity through quantitative measurement of liver stiffness [55]. However, research has found that the completely automated DL algorithm based on gadoxetic acid-enhanced MRI shows no significant difference in AUC values compared to MRE at any LF phases (*p* > 0.134) [46]. This comparative study indicates that the diagnostic effectiveness of DL and MRE is comparable, DL might allow for fibrosis staging in hospitals that do not have specialized MRE hardware/drivers. Further studies are needed on the combined evaluation performance of MRE and DL in LF lesions. In addition to these conventional sequences, in recent years, molecular imaging has become a cutting-edge field for evaluating LF. Researchers have developed a single-chain peptide probe (F-GhypO) that specifically targets collagen in lesions for the precise staging of LF [56]. This probe forms a unique monomeric conformation by replacing proline with (2S,4S)-hydroxyproline (hyp) and subsequently disrupting hydrogen bonds, thereby showing a higher selectivity for pathological collagen. In the mouse fibrosis model, T1-weighted MRI shows that, with the injection of Gd-GhypO, the signal enhancement is consistent with the severity of fibrosis, and the contrast-to-noise ratio (ΔCNR) increases accordingly. Additionally, biodistribution analysis indicates that gadolinium residue in major organs 24 h post-injection is relatively low, ensuring the safety of this probe in molecular imaging.

Moreover, multi-sequence fusion and DL are important directions for improving LF evaluation performance. A study proposed an automatic evaluation of liver cirrhosis staging based on a DL framework using multiple MRI sequences. This framework combines multi-scale feature learning with specific sequences to capture subtle tissue changes between different stages of cirrhosis progression. The best model achieved accuracies of 72.8% and 63.8% on T1WI and T2WI, respectively, significantly outperforming traditional imaging radiomics methods [57]. In summary, multi-sequence MRI, by combining conventional sequences, functional imaging and emerging molecular imaging technologies, provides a comprehensive evaluation platform for LF. With the integration of AI technologies, the application value of MRI in non-invasive diagnosis, precise staging and prognostic prediction of LF will be further enhanced, offering more accurate decision support for clinical practice.

### 3.2. CT Imaging Technology

Although MRI has become the preferred reference standard for quantifying LF, CT plays a crucial role in incidentally detecting undiagnosed illnesses owing to its rapid scanning capabilities and superior spatial clarity, and it offers broader application possibilities [58].

CT-based evaluation of LF primarily relies on the texture characteristics, morphological changes and hemodynamic alterations of the liver parenchyma [59]. As fibrosis progresses, CT images reveal a blunted liver margin, a nodular surface, and coarse texture [60]. Perfusion CT (pCT) involves acquiring a series of high temporal resolution images after intravenous injection of a high-concentration iodinated contrast agent. Subsequent image processing allows for the calculation of quantitative or semi-quantitative tissue perfusion parameters (e.g., blood flow, blood volume, mean transit time, portal venous liver perfusion, arterial liver perfusion and liver perfusion index) [61], thereby quantifying the fibrotic morphological changes in liver disease.

The potential of DL-based CT image analysis in achieving liver disease tissue segmentation and quantitative analysis has been proven [59]. According to relevant research reports, DL can use CT-based data drawn from the liver surface, parenchyma and extrahepatic data for forecasting the LF stages [44]. These researchers used portal phase enhanced CT images from 252 patients who were pathologically confirmed to have LF, successfully constructing a fibrosis staging network. By utilizing gradient-weighted class activation mapping to generate location maps, they highlighted the focal areas of the LF staging network when predicting the stages of fibrosis. Their findings demonstrated that the network yielded AUC values of 0.92 (significant fibrosis, F2–F4), 0.89 (advanced fibrosis, F3–F4) and 0.88 (cirrhosis, F4) in the corresponding assessments. Additionally, it was noted that for F0 patients, the focus is more on the liver surface, whereas for F4 patients, the emphasis shifts towards the parenchymal regions of the liver and spleen. This confirms that the DL algorithm can stage LF through contrast-enhanced CT images, providing a standardized tool for its quantitative evaluation.

In view of the large volume of CT data, there is a growing interest in bridging the gap between imaging and textual reports. While neural networks are actively used for generating medical findings in chest X-rays, similar applications for LF are still in the exploratory stage due to the complexity of 3D abdominal volumes. However, foundation models are paving the way. Researchers have developed a key-slice selection framework based on contrastive language-image pretraining (CLIP), enabling each sentence in a radiology report to be matched with its most informative CT slice [62]. By leveraging text-image similarity matching, this method not only supports medical visual question-answering (CLIA) but also lays the technical foundation for future automatic report generation and the description of LF features. Further research is needed to evolve from slice retrieval to generating comprehensive descriptive findings for fibrosis staging, thereby improving evaluation efficiency. In addition, performing organ segmentation in CT scans aids in diagnosing, treatment planning and executing therapeutic procedures [63]. DL-based segmentation algorithms that require no prior registration enable higher accuracy in multi-organ abdominal CT scans, thereby playing a role in image-guided navigation [64]. Zheng and colleagues developed a DL-based automated segmentation and classification model, finding that its performance (AUC 0.89–0.92) surpassed models based on the liver (AUC 0.84–0.90) or spleen (AUC 0.69–0.70). Through test-time adaptation, the model’s Obuchowski index values across three external datasets increased from 0.81, 0.73 and 0.73 to 0.85, 0.85 and 0.81, enabling highly accurate diagnosis of LF [65]. This also demonstrates the potential of real-time fine-grained organ segmentation in clinical applications.

### 3.3. Ultrasound Imaging Technology

Driven by its multiple advantages, such as being simple, repeatable, radiation-free, providing real-time imaging and being relatively low in cost, ultrasound examination has become the preferred screening modality for liver disease screening [66]. Conventional liver ultrasound can assess morphological indicators such as organ shape, capsule smoothness, actual echo intensity/uniformity and vascular clarity, but its sensitivity to early LF is limited [67].

Emerging ultrasound elastography encompasses various techniques including strain elastography and shear-wave elastography [68]. These methods have been widely applied to measure liver stiffness, thereby assessing the stage and severity of LF [69]. As LF progresses, liver stiffness values increase, which markedly enhances the diagnostic value of ultrasound for LF. In addition, DL technology has also demonstrated strong potential in ultrasound image analysis. Multiple studies have shown that DL models can extract information from ultrasound images, enabling a more accurate characterization of the degree of LF [26,70]. Chen et al. [71] developed an ultrasound-based DL model, where this fibrosis ultrasound DL network was trained to predict whether the liver imaged might have a stiffness greater than or equal to 8.7 kPa, thereby determining the presence of severe fibrosis. They found that the model performed better in diagnosing advanced LF with higher accuracy. Therefore, combining DL methods with ultrasound radiomics seems to augment the precision of ultrasound imaging technology for the preliminary assessment and quantification of LF.

In addition, ultrasound-guided microscopy is a non-invasive technique capable of imaging microvasculature within the body. It achieves imaging resolution by locating and tracking the microbubble trajectory injected into the blood flow of mice [72]. The trade-off between microbubble concentration and positioning precision and accuracy can be partially mitigated through DL-based methods [73]. Experts have proposed sparse tensor neural networks to realize a DL-based 3D ultrasound localizing microscope, enhancing memory scalability through dimensionality expansion. In the 3D mode, this approach reduces memory requirements by two orders of magnitude while significantly outperforming traditional ultrasound positioning microscopes in high-concentration settings. This technology offers new possibilities for providing information on LF-related microcirculation changes and for its evaluation. However, it should be noted that the technology involving super-resolution imaging through microbubble tracking remains in the research phase and requires further studies and clinical validation to determine its feasibility and effectiveness in human clinical settings.

In summary, multimodal imaging technologies provide a comprehensive evaluation platform for LF by combining their unique strengths. A promising current research direction involves combining AI with complementary datasets derived from multiple imaging modalities to identify pathologies and diseases. With the further integration of AI technologies such as DL and multimodal data involving imaging and biomarkers, LF’s capabilities in non-invasive diagnosis, precise staging and prognostic prediction will be significantly enhanced, providing more accurate decision support for clinical practice. A comparative summary of representative studies and their performance metrics across these imaging modalities is presented in Table 2.

## 4. Clinical Application Scenarios

Radiomics and DL technology have demonstrated exceptional performance in the precise assessment of LF diagnosis and disease staging, with relevant studies having been discussed earlier. The subsequent sections will be dedicated to analyzing and discussing the significance of these technologies in the prognosis assessment and mechanistic exploration of this disease.

### 4.1. Prognostic Prediction and Risk Stratification

Prognosis prediction and risk stratification for LF are crucial for clinical treatment decisions and long-term patient management [74]. With the development of AI technology, prognostic prediction systems based on imaging genomics, digital pathology and multi-parameter models have significantly enhanced the ability to predict clinical outcomes in LF patients [75].

The remarkable value of MRI radiomics in predicting disease prognosis has attracted significant attention in recent years: a study on hepatitis B virus-related fibrosis suggested that MRI-based radiomics can effectively predict the risk of liver-related events including ascites, variceal bleeding, hepatorenal syndrome, hepatic encephalopathy, and hepatocellular carcinoma [23]. This study analyzes the most predictive features through SHapley additive explanations (SHAP), reflecting the heterogeneity in the distribution of internal liver fibrosis tissue, thereby providing a basis for decision-making in the hepatitis B-related liver diseases treatment. Recently, digital pathology has, likewise, made significant advances in predicting the LF prognosis [76]. Louis et al. designed a novel phenotypic digital pathology platform to assess fibrosis severity and sensitivity to sample variability. The study found that phenotypic digital pathology biomarkers provide reliable and continuous measurements of fibrosis severity and activity. By enhancing resolution and reducing sensitivity to biopsy variability, they offer prognostic information beyond traditional histological scoring [77], providing new tools for precision management of LF.

In addition, multiparametric machine learning models have also shown strong prognostic predictive value in patients with LF [78]. Luo et al. developed a radiomics model using liver MRI to predict the risk of liver-related events in patients with chronic hepatitis B virus-induced fibrosis undergoing antiviral therapy. This model was developed using a support vector machine classifier and demonstrated good calibration, as evidenced by AUCs of 0.94 (training set) and 0.93 (test set) [23]. This study proves that machine learning techniques are crucial for evaluating disease progression, prognosis prediction and initiating treatment among LF and hepatitis patients. Similarly, DL technology has also shown prognostic value in ultrasound imaging analysis: some researchers have pointed out that the CNN model based on stacked microvascular imaging achieved an accuracy of 83.8% in distinguishing significant LF (≥F2), which is higher than the 81.6% accuracy assessed by the examiners’ scoring [79]. This indicates that DL technology not only provides objective fibrosis analysis but also eliminates human subjective biases, enabling more reliable risk stratification for LF progression.

### 4.2. Etiological Differentiation and Mechanism Exploration

The etiologies of LF are diverse and its pathogenesis is complex, making accurate differentiation of the underlying causes and in-depth exploration of the molecular mechanisms crucial for personalized therapy [80]. The integration of radiomics and DL technologies offers a new breakthrough for understanding the distinctive etiological features and molecular mechanisms of LF.

In research targeting LF caused by protein misfolding diseases, researchers employed mass spectrometry-based spatial proteomics technology combined with machine learning methods to create the first high-resolution map of protein toxicity processes within human liver tissue [81]. They found that in LF caused by α1-antitrypsin deficiency, the peroxisome-associated protein in hepatocytes was significantly upregulated at the early stage of α1-antitrypsin accumulation, preceding the classic unfolded protein response. More importantly, the peroxisomal response was more pronounced in patients at the low fibrosis stage, while it was significantly delayed at the high fibrosis stage. This discovery suggests that peroxisome function could be a key target for early intervention and provides new ideas for targeted treatment strategies. In addition, the research team from the Shanghai Institute of Materia Medica of the Chinese Academy of Sciences, in collaboration with the Lingang Laboratory, has constructed the world’s first high-precision, complete 3D liver pathological atlas, offering a fresh perspective on the spatial distribution characteristics of LF [82]. The research team employed DL technology to automatically segment and analyze large-scale three-dimensional pathological image datasets. Through training on extensive image data, the DL model achieved automated identification and segmentation of fatty liver cells, precisely calculating their volumetric proportion. Results revealed that the volume proportion of fatty liver cells in LF mouse livers reached 30.76%. Concurrently, the study found relatively consistent fatty degeneration across different hepatic lobes, further confirming the prevalence and regional specificity of LF pathological alterations. Beyond offering fresh perspectives on LF pathogenesis, this also provides precise coordinates for potential target discovery and targeted therapy. Additionally, AI has demonstrated potential in distinguishing LF caused by different etiologies [83]. In the future, AI technology can be used to integrate digital pathology with DL to further explore mechanisms and targets.

In summary, the integration of multimodal technologies not only demonstrates superior performance in the prognostic prediction and risk stratification of LF, but also provides research directions for etiological differentiation and mechanism exploration. These advancements lay a solid foundation for the cyclical management and clinical treatment of LF.

## 5. Summary and Outlook

### 5.1. Current Challenges and Limitations

Although radiomics and DL have demonstrated significant potential in assessing LF, these technologies still face numerous challenges in transitioning from studies to clinical practices [84]: Firstly, technical reproducibility and standardization represent one of the primary challenges currently faced. Given that radiomics features are susceptible to various factors such as image acquisition and reconstruction methods, images obtained from the same patient using different scanners or parameters may yield significantly divergent feature values. This severely compromises the models’ generalization capability [85]. Therefore, establishing a standardized image acquisition protocol and systematic feature extraction workflow for this problem—including image preprocessing, ROI segmentation, feature extraction and model validation—is a prerequisite [86]. The introduction of tools like LIFEx can mitigate this issue to some extent. By providing standardized and user-friendly workflows across different imaging modalities, it addresses heterogeneity in upstream steps such as image acquisition, reconstruction and ROI segmentation. This enhances the accessibility, transparency and reproducibility of radiomics feature extraction [87]. Similarly, DL models also face challenges related to reproducibility. Deep neural networks are highly sensitive to the distribution of input data, and data imbalance during training and testing, as well as overfitting, may severely compromise model reliability and performance [88]. Hence, developing robust models that are insensitive to technical factors will be an important direction for future research.

Secondly, the development of robust models faces a dual challenge regarding data availability and reliability [89]. Unlike lung pathologies where open-source datasets are accessible, publicly available datasets with direct download links for LF staging are currently scarce due to the invasive nature of the gold-standard biopsy. Consequently, most models rely on private, single-center cohorts, restricting external validation. Furthermore, the “ground truth” derived from biopsy is imperfect, suffering from sampling errors and inter-observer variability. Training on such noisy labels can destabilize model convergence and lead to inflated performance metrics that do not reflect real-world utility [90]. To mitigate the impact of imperfect ground truth, future research must move beyond simple supervision and explore strategies such as consensus labeling by multiple pathologists, uncertainty-aware modeling, and robust loss functions designed for noisy data [91]. Additionally, leveraging composite reference standards or emerging resources like the high-precision 3D liver pathological atlas may help overcome the limitations of traditional biopsy sampling [82]. Thirdly, even with the implementation of specific optimization strategies, the generalization capability of the constructed model could be restricted, primarily attributable to the limited number of samples [92]. Thus, prospective investigations are necessary to validate the model’s performance and enhance its generalizability, which is key to advancing its clinical application. Additionally, how to seamlessly integrate imaging genomics and DL tools into clinical workflows to provide intuitive and easily understandable results presentation and clinical decision support is also an area that requires attention in the future.

### 5.2. Future Outlook

LF represents a multifaceted pathological process involving alterations at multiple levels, including various molecules, cells, and organ tissues [2]. As technology progresses and clinical validation strengthens, the prospects for the application of radiomics and DL in LF assessment are promising [93]. First, single-modality data struggles to fully capture this complexity, and future research will increasingly focus on multimodal data fusion [94]. By integrating multidimensional information from various imaging modalities (MRI, CT and ultrasound), clinical indicators, genomics and proteomics, we aim to establish a more comprehensive LF assessment system. Second, the “black box” nature and lack of interpretability of DL models are key factors limiting their clinical application [93]. Therefore, developing explainable AI technologies is a principal pathway for upcoming studies. To advance the clinical translation of radiomics and DL technologies, standardized workflows must be established and rigorously validated through prospective studies [95]. This includes standardized image acquisition protocols, feature extraction processes, model development and validation methodologies to enhance research quality and comparability. Concurrently, large-scale multicenter prospective investigations are essential to provide the strongest evidence supporting the clinical adoption of these technologies. Finally, with advances in computing technology and the refinement of AI policies, radiomics and DL technologies will gradually spread from leading research institutions to general medical facilities. This expansion will enable more healthcare providers to benefit, helping to increase the early screening rate for LF and achieve early diagnosis and treatment.

## 6. Conclusions

Radiomics and DL technologies are gradually transforming the evaluation of LF. By extracting non-visualizable quantitative features from conventional medical images and combining them with powerful DL algorithms, these technologies enable more accurate diagnosis and staging of LF, prognosis prediction and even exploration of underlying mechanisms. The significant advantage of DL lies in its ability to automatically learn complex patterns from raw image data, thereby enhancing diagnostic accuracy and supporting large-scale non-invasive assessment. However, limitations exist, such as the requirement for massive datasets, the “black box” nature of models and potential issues like overfitting and data bias. To achieve widespread clinical adoption of DL, these challenges must be addressed. Furthermore, issues such as standardization, reproducibility, generalization capability and clinical integration remain major obstacles. Therefore, future research should focus on multimodal data fusion, improving AI interpretability, establishing standardized workflows and conducting large-scale prospective validation. As these challenges are progressively overcome, such technologies hold promise to become core tools for precision medicine in LF, ultimately improving the clinical outcomes for patients with chronic liver disease.

## Figures and Tables

**Table 1:** Comparison of Feature Characteristics: Radiomics vs. Deep Learning.

Category	Radiomics	Deep Learning
Generation Mechanism	Feature Engineering: Mathematically defined formulas designed by experts to quantify specific patterns (e.g., heterogeneity).	Feature Learning: Automatically discovered hierarchical representations via backpropagation, optimized for the specific task.
Feature Complexity	Explicit & Fixed: Ranging from simple morphological (shape, volume) to complex transformed features (Wavelet, Laplacian of Gaussian).	Hierarchical & Abstract: Progressing from low-level edges/textures to high-level semantic patterns (e.g., nodular surface, distorted boundaries).
Interpretability Status	Mixed Interpretability:	Evolving Interpretability (XAI):
	High for morphological/first-order features (intuitively linked to pathology). Low for high-order features (e.g., Wavelet-based textures), which are often abstract and lack direct biological translation.	Traditionally considered a “Black Box” due to nonlinear complexity. Currently improved by Explainable AI methods (e.g., Grad-CAM attention maps, SHAP values) to visualize decision focus.
Workflow Dependency	Heavily dependent on precise ROI segmentation and standardization of image acquisition/reconstruction.	Less dependent on manual segmentation (end-to-end); can utilize whole-volume inputs but requires large-scale data for robustness.

**Note:** XAI = Explainable Artificial Intelligence; ROI = Region of Interest; Grad-CAM = Gradient-weighted Class Activation Mapping; SHAP = SHapley Additive exPlanations.

**Table 2:** Summary of Representative Studies on Radiomics and Deep Learning for Liver Fibrosis Assessment.

Modality	Reference	Method	Algorithm/Model	Task	Key Performance (Validation/Test)
MRI	Luo et al. [23]	Radiomics	SVM	Risk prediction (Liver-related events)	AUC: 0.93
	Cunha et al. [29]	Deep Learning	CNN	Fibrosis staging	AUC: 0.89–0.93 (Comparable to manual)
	Yasaka et al. [51]	Deep Learning	DCNN	Fibrosis staging	AUC: 0.85 (F2), 0.84 (F3), 0.84 (F4)
	Zhu et al. [54]	Deep Learning	5-layer DCNN	Fibrosis Staging (Chronic Hepatitis B)	Accuracy: 88.13%
CT	Wang et al. [22]	Radiomics	Machine Learning	Fibrosis staging	AUC: 0.84–0.90
	Yin et al. [44]	Deep Learning	DL Network	Fibrosis staging	AUC: 0.92 (F ≥ 2), 0.89 (F ≥ 3), 0.88 (F4)
	Zheng et al. [65]	Deep Learning	Segmentation & Classification	Organ segmentation & Staging	AUC: 0.89–0.92
Ultrasound	Zhang et al. [30]	Deep Learning	DL Model	Staging (High-frequency US)	Accuracy: 0.93 (Advanced fibrosis)
	Zhan et al. [32]	Deep Learning	AutoFibroNet	Fibrosis quantification	AUROC: 0.94 (F4), 0.80 (F3), 0.83 (F2)
	Xue et al. [33]	Radiomics	Transfer Learning	Grading	AUC: 0.950 (S4), 0.932 (≥S3), 0.930 (≥S2)

**Note:** MRI: Magnetic Resonance Imaging; CT: Computed Tomography; US: Ultrasound; CNN: Convolutional Neural Network; DCNN: Deep convolutional neural networks; DL: Deep Learning; AUROC: Area Under the Receiver Operating Characteristic Curve; SVM: Support Vector Machine; AUC: Area Under the Curve.

## Data Availability

No new data were created or analyzed in this study. Data sharing is not applicable to this article.
